# Oxytocin Receptor-Mediated Astrocytic Signalling: Novel Anxiolytic Mechanisms and Implications for Social Cognition Disorders

**DOI:** 10.1192/j.eurpsy.2026.10728

**Published:** 2026-07-31

**Authors:** A. H. I. Abu Shehab, A. B. Ciubară, Ș. L. Burlea, A. Lucreția, I. Chiscop, A. Ciubară

**Affiliations:** 1 Psychiatry, “Elisabeta Doamna” Psychiatry Hospital, Galați; 2 Orthopedics & Traumatology, “Sf. Apostol Andrei” Emergency Clinical County Hospital, Galați; Faculty of Medicine & Pharmacy, “Dunărea de Jos” University of Galați, Galati; 3 Psychiatry, “Grigore T. Popa” University of Medicine and Pharmacy (UMF), Iași, Iasi; 4 Internal Medicine; 5 Oral & Maxillofacial Surgery, “Sf. Apostol Andrei” Emergency Clinical County Hospital, Galați; Faculty of Medicine & Pharmacy, “Dunărea de Jos” University of Galați; 6 Psychiatry, Faculty of Medicine and Pharmacy, “Dunărea de Jos” University of Galați; “Elisabeta Doamna” Psychiatry Hospital, Galați, Galati, Romania

## Abstract

**Introduction:**

Oxytocin, a neuropeptide traditionally associated with social bonding and reproductive functions, has emerged as a critical modulator of anxiety and social cognition. Recent paradigm-shifting research has revealed that astrocytes, previously considered passive support cells, are essential mediators of oxytocin’s anxiolytic effects through novel signaling pathways distinct from neuronal mechanisms.

**Objectives:**

To synthesize current understanding of oxytocin receptor-mediated astrocytic signaling mechanisms, examine their role in anxiolysis, and evaluate therapeutic implications for social cognition disorders including autism spectrum disorder, schizophrenia, and ADHD.

**Methods:**

Comprehensive literature review of peer-reviewed publications from major databases including PubMed, Nature, and specialized psychiatric journals. Analysis focused on molecular mechanisms of oxytocin receptor signaling, astrocytic pathways, anxiolytic mechanisms, and clinical applications in social cognition disorders.

**Results:**

Oxytocin receptor activation in astrocytes triggers a novel Sp1-Gem signalling cascade, distinct from neuronal pathways. This mechanism involves transcription factor Sp1 upregulation, increased Gem expression, and subsequent RhoA/ROCK pathway inhibition, leading to cytoskeletal remodelling and process elongation. Astrocytic oxytocin signalling modulates gap-junctional communication through connexin regulation and alters astrocyte-synapse spatial relationships. Astrocyte-specific knockdown of oxytocin receptors or Gem completely abolishes oxytocin’s anxiolytic effects, establishing astrocytes as necessary mediators. Clinical evidence demonstrates oxytocin’s therapeutic potential in autism spectrum disorder, with intranasal administration improving social cognition and reducing repetitive behaviors.

**Conclusions:**

The discovery of astrocytic oxytocin signaling represents a fundamental paradigm shift in understanding anxiolytic mechanisms. The Sp1-Gem pathway offers novel therapeutic targets for anxiety and social cognition disorders, potentially enabling more precise interventions than global oxytocin administration. This research opens new avenues for treating neuropsychiatric conditions through astrocyte-specific modulation.

**Disclosure of Interest:**

None Declared

